# What is it going to take to move youth-related HIV programme policies into practice in Africa?

**DOI:** 10.7448/IAS.20.4.21491

**Published:** 2017-05-16

**Authors:** Daniella Mark, Lina Taing, Lucie Cluver, Chris Collins, Kate Iorpenda, Catarina Andrade, Luann Hatane

**Affiliations:** ^a^ Paediatric-Adolescent Treatment Africa (PATA), Cape Town, South Africa; ^b^ Department of Psychology, University of Cape Town, Cape Town, South Africa; ^c^ Department of Social Policy and Intervention, University of Oxford, Oxford, UK; ^d^ Department of Psychiatry, University of Cape Town, Cape Town, South Africa; ^e^ Friends of the Global Fight, Washington, DC, USA; ^f^ Independent Consultant, Brighton, England

**Keywords:** Transition, HIV, adolescent, policy, practice, guideline

## Abstract

**Introduction**: HIV has been reported to be the leading cause of mortality amongst adolescents in Africa. This has brought attention to the changes in service provision and health management that many adolescents living with HIV experience when transferring from specialized paediatric- or adolescent-focused services to adult care. When transition is enacted poorly, adherence may be affected and the continuum of care disrupted. As the population of HIV-infected adolescents grows, effective and supported transition increases in significance as an operational imperative.

**Discussion**: Considerable gaps remain in moving policy to practice at global, national, and local levels. Policies that give clear definition to transition and provide standard operating procedures or tools to support this process are lacking. National guidelines tend to neglect transition. Beyond transition itself, there has been slow progress on the inclusion of adolescents in national policies and strategies. Guidance often overlooks the specific needs and rights of adolescents, in particular for those living with HIV. In some cases, prohibitive laws can impede adolescent access by applying age of consent restriction to HIV testing, counselling and treatment, as well as SRH services. Where adolescent-focused policies do exist, they have been slow to emerge as tangible operating procedures at health facility level. A key barrier is the nature of existing transition guidance, which tends to recommend an individualized, client-centred approach, driven by clinicians. In low- and middle-income settings, flexible responses are resource intensive and time consuming, and therefore challenging to implement amidst staff shortages and administrative challenges. First, national governments must adopt transition-specific policies to ensure that adolescents seamlessly receive appropriate and supportive care. Second, transition policies must form part of a broader adolescent-centred policy landscape and adolescent-friendly orientation and approach at health system level. Third, national actors must ensure that transition policies are supported at implementation level. Fourth, youth involvement and community mobilization are essential. Finally, further implementation research is urgently needed to better understand how to support young people and providers in achieving smooth transitions.

**Conclusions**: Only by moving from policy to practice through supportive policies and their implementation will we be closer to including adolescents in the 2030 goal of ending AIDS.

## Introduction

Global HIV/AIDS targets refer to ending the AIDS epidemic by 2030 [1]. Evidence suggests, however, that this optimism does not hold for young people. There are 29 new HIV infections among adolescents aged 15–19 every hour globally [2]. It is estimated that HIV-related deaths among adolescents living with HIV (ALHIV) have tripled since 2000, making HIV one of the leading causes of mortality in this age group in Africa [3]. As young people reach adulthood, those who have been receiving specialized paediatric/adolescent services must be transferred to adult care and begin managing their own health. Adolescent transitioning within an HIV treatment and care context is understood as a shift from child-focused facilities and/or staff to adult-focused health services, as well as increasing self-management, where the responsibility for personal health shifts from health providers and caregivers to adolescents themselves [4,5]. Failure to manage this transition effectively can disrupt the continuum of care and negatively impact treatment outcomes [6,7].

As the population of HIV-infected adolescents grows, effective and supported transition increases in significance as an operational imperative. For the first time, WHO recommended the implementation of adolescent-friendly health services (AFHS) in HIV services in the second edition of its consolidated guidelines for treating and preventing HIV infection [8]. Despite this and other efforts, implementation of AFHS that are integrated, standardized, coordinated and of high quality are lacking globally, and in sub-Saharan Africa in particular. This commentary reviews gaps and clarifies what is required to move youth-related HIV policy into practice in order to ensure that young people living with HIV can also live long and healthy lives into 2030 and beyond.

## Discussion

A renewed focus on ALHIV is evidenced by global efforts – such as All-in, DREAMS, Start Free, Stay Free, AIDS Free [9–11] – and youth-friendly normative guidance and national policies, including those that impact transition directly and indirectly. The WHO consolidated guidelines [8] refers to the establishment of linkages and referral pathways to ensure a comprehensive continuum of care, especially during transition. Considerable gaps remain, however, in moving policy to practice at global, national and local levels.

### Policy gaps at national level

Policies that provide clear definitions, and standard operating procedures and tools to support transition are lacking. With the exception of a few countries – which provide clear guidance on when and how to transition [4,12–14] – national guidelines tend to neglect transitional age and process. In fact, of the six countries in which half of all HIV-positive adolescents aged 15–19 years reside worldwide (South Africa, Nigeria, Kenya, India, Mozambique, and Tanzania), Kenya is the only country whose national ART guidelines provides advice on adolescent transition [15–18]. Addressing policy oversights in adolescent-centred sexual and reproductive health (SRH); mental health; peer to peer services; and family-centred psychosocial care may build ALHIV resilience and provide social protections to mitigate structural barriers hindering access and retention in care.

Prohibitive laws can impede adolescent access by applying age of consent restriction to HIV testing, counselling and treatment, as well as SRH services. Muller and colleagues [19] have observed the contradictory obligations of South African law which protects teenagers’ rights to make decisions regarding reproduction – allowing them to consent to receiving contraception at the age of 12 and giving girls at any age the right to terminate a pregnancy, but imposing an age of sexual consent at 16 years. Furthermore, a number of countries – for example, Uganda, Tanzania, and Zambia – criminalize same sex relationships [20], drug use, the selling of sex and HIV transmission, which perpetuates discrimination and undermines access to critical services for ALHIV from key populations. This poses additional complexities and dilemmas for service providers who are required to navigate policy limitations whilst meeting an ethical imperative to safeguard the rights of young people and ensure they are provided information, services and support. These policy omissions, contradictions and restrictions mean that critical services and support that could effectively prepare adolescents for self-management after the transition period are missing from national health strategies.

### Procedural gaps at facility level

Where adolescent-focused policies do exist, they have been slow to emerge as tangible operating procedures at health facility level. There are multiple challenges that impede implementation. A key barrier is the nature of existing transition guidance and programmatic protocols [21,22] which tend to emanate from the United States. Typically, a highly client-centred approach, driven by clinicians and tailored to the individual adolescent is advised [21,23,24]. Recommendations also suggest a phased process based on adolescent developmental readiness, with careful planning and a minimum package of services that addresses the full spectrum of adolescent clinical and psychosocial needs. This is best practice due to the way HIV impacts development and because of the unique needs of ALHIV infected vertically and those infected horizontally. However, such flexible and comprehensive responses are resource intensive, making them challenging to implement in low- and middle-income settings amidst staff shortages and administrative challenges. Thus, within low- and middle-income country contexts, effective transition as recommended is often limited to heavily donor-resourced programmes.

### Implementation barriers at health facilities

When transition protocols are in place, it is common for them to be overlooked. For example, the capacity of health facilities to implement these protocols can be undermined by staff shortages, absenteeism, and turnover [14], especially in low- and middle-income settings [25]. Resources to train and supervise staff on new protocols [14] are insufficient. Adolescence is a period of rapid physical and emotional change, but services rarely accommodate the neurocognitive changes that are ongoing in adolescence, where executive functions – such as planning, working memory and impulse control – are still maturing [26]. At the individual level, adolescents who have grown up within ART services are assumed to have a thorough understanding of HIV and the role of treatment. However, it is clear that many adolescents have gaps in understanding [27] that directly impact adherence. Given less supervision and personalized support in adult services, transition is often resisted by the health provider, adolescent and/or caregiver in order to retain intensive, individualized care, rather than progress to adult services which are generally more fragmented and provide less support [28].

Health provider stigma is also a central implementation barrier in the provision of adolescent care. For example, staff report moral dilemmas, anger towards adolescent patients, and a concern that discussing sexual activity or providing contraception is “permitting” risky activity [29].

### Gender-specific implementation barriers

Adolescent girls face higher risk of HIV infection and continue to be disproportionately affected by HIV, in particular in sub-Saharan Africa where they account for three in four new infections among 15–19 year olds [2]. However, adolescent girls face unique barriers to accessing AFHS and transition support. For instance, pregnant teenage girls in paediatric or adolescent services – including those who fall pregnant as a result of rape – are often transitioned into adult services, regardless of age or readiness. Young girls living with HIV are less likely to access appropriate services for the prevention of mother-to-child transmission of HIV (PMTCT) [30], and premature transitioning will likely limit this ability further as they receive less intensive and individualized monitoring, care, and adherence support in adult services. This reduction in support within adult services can have a number of implications for adolescent girls who are already experiencing poor adherence to ART during pregnancy, and increased risk of treatment failure and vertical transmission.

### Gaps in community, family, and social support

Adolescence does not happen primarily within the clinic, and so it is essential to identify barriers to transition and engagement in care at the family, community and social levels [31]. Family engagement in supporting ALHIV during periods of transition influences care and treatment outcomes. Research shows that some responses to HIV that aim to protect children can become harmful to adolescents [32]; for example, adolescents who have not been disclosed their HIV status have three times the odds of non-adherence to ART [33]. Mental health distress is also associated with difficulty in accessing healthcare [34]. Studies in the US suggest that this may be closely related to the family and community context rather than HIV infection itself [35,36].

At a community and social level, evidence suggests [37] that adolescents and young people living with HIV face additional and more complex challenges as they explore their sexual identity, form relationships and face disclosure to partners and peers. Qualitative studies identify stigma as a central barrier for adolescents as they struggle to maintain treatment, and particular challenges in managing adherence and contraception within shifting and often fraught teenage romantic relationships [38]. Coupled with this are the challenges of understanding options for effective contraception while on ART, navigating safe sex, safer conception and mitigating the risks of mother-to-child transmission. Adolescence is in itself a period of transition, and ALHIV are exploring sexuality and gender identity. Social stigma associated with these may result in many young people hiding behaviours and not seeking information, health services and support. Exclusion from, and discrimination by communities further exposes ALHIV to increased risk of violence, family separation, isolation and mental health problems.

### Limited evidence for service delivery interventions

There remains a lack of evidence for the effectiveness of service delivery interventions to support ALHIV within healthcare settings, including interventions aimed at supporting transition periods. One systematic review [39] noted that available evidence comes from a small number of studies with low to moderate methodological quality. The absence of evidence and operations research limits the ability of programmes and governments in low- and middle-income settings to prioritize interventions and determine their cost-effectiveness. It also prevents monitoring impact of interventions on the health system, for example managing the risk of task shifting to lay workers.

### Recommendations for moving transition and youth-related HIV policy into practice

Several recommendations on moving policies into practice can be made and these are captured in Box 1. These recommendations are still relevant for settings where adolescents receive care in primary-care clinics and no transition from paediatric to adolescent or adult care occurs.

**Box 1.** Moving youth-related HIV policies into practice: 5 key components

National governments adopt transition-specific policies to ensure that adolescents receive appropriate and supportive treatment and care Transition policies form part of a broader adolescent-centred policy landscape and adolescent-friendly orientation and approach at health system level National actors ensure that transition policies are supported at implementation level, and that transition-related activities are provided for in national health budgets Youth involvement and community mobilization are incorporated into implementation models Further implementation research is carried out to better understand how to support young people and providers in achieving smooth transitions

First, national governments adopt transition-specific policies to ensure that adolescents seamlessly receive appropriate and supportive care. These policies should establish a clear definition of transition, with transitional age, the necessary elements of an effective transition processes, and stakeholder roles described. Guidance on retaining pregnant teenagers in paediatric/adolescent programmes with PMTCT support is especially needed. Policies should be situated within national strategic plans, with clear recommendations and quality standards. These policies should be applicable within low- and middle-income contexts, where the HIV burden is heaviest. Perhaps most helpful to adopt is a public health approach which acknowledges the widespread and urgent need for services and accommodates it with a simplified approach to care [23]. In addition, specific legal changes may be necessary to allow young people full access to services and engagement in care. A key example is lowering the age of consent for HIV testing and care services.

Second, transition policies form part of a broader adolescent-centred policy landscape and adolescent-friendly orientation and approach at health system level. HIV services in general should be appropriate and tailored to the unique and diverse needs of ALHIV to build resilience before transition. Young people require a comprehensive, integrated package of AFHS that responds to their particular needs and are equitable, accessible, acceptable, appropriate and effective [40]. This package should integrate HIV treatment and care, psychosocial support and SRH services.

Of central importance are national policies that ensure stigma-free services for all young people. Specific interventions are needed to alleviate stigma towards young key populations. This includes health worker training to improve competencies in adolescent care as well as shifting health worker attitudes towards adolescents and their right to care [41]. The WHO has prepared comprehensive guidelines for HIV services for young key populations, including sexual and reproductive health and mental health services [8]. Policies should aim to also ensure comprehensiveness of services, particularly for young people who are poor or socially marginalized. Social protection measures may need to be considered to address structural drivers of vulnerability. Financial and other supports can encourage school enrolment and continued school participation. There are also calls for broader health system reforms and policy changes that transform care, planning and service delivery so that it is informed by a developmental perspective and more fully addresses the contextual factors in care access and wellness.

Third, national actors ensure that transition policies are supported at implementation level. Formal transition protocols and standard operating procedures are necessary, as are tools based on emerging evidence of effective implementation models. Moreover, transition-related activities should be provided for and earmarked in national health budget lines. Educational institutions and professional associations must provide staff training, sensitization and continued professional development. Policies should provide for monitoring and reporting systems that measure, track, and evaluate transition milestones, with established indicators as means to assess transition outcomes. Communication and linkage between paediatric and adult points of care must be improved. A multisector approach may be used to address social, economic, legal and other structural barriers that limit access by leveraging international and local non-governmental organizations, faith-based organizations, community structures, and schools to reduce loss to follow-up during the transition period. Investments in both systems infrastructure and local implementation processes are needed to overcome operational barriers [42].

Fourth, youth involvement and community mobilization are essential. Young people and their communities need to be actively involved in addressing healthcare issues that affect them to improve the uptake of services, programme quality and accountability. Governments cannot expect to deliver successful transition services without listening to the needs expressed by young people themselves, and including them in design and evaluation. It is necessary to coordinate linkages and referrals to communities for continuity of support during and after transition. Adolescence sees increased importance of peer relationships and reduced family influence. This growing autonomy is an important consideration in developing appropriate support for adolescents that can draw on the power of peers and determine what role families continue to play. Recognizing and integrating youth living with HIV as lay workers and counsellors, for example AfricAid’s Zvandiri Programme, may help peers to navigate the health system and share positive coping strategies [43]. Families and caregivers should be sensitized to understand the importance of support during transition and be provided with information on how to actively take part in and support ALHIV in times of transition and growing autonomy. Many ALHIV deal with bereavement, treatment fatigue, and finding a sense of purpose and hope for the future. The provision of family and community support is critical for psychosocial wellbeing during the transition process. There is need to reinforce good disclosure processes particularly with families and provide guidance in onward disclosure.

Community support mechanisms, and the strategic engagement of community-based organizations as key partners in service provision is critical. New data from South Africa show that combinations of social, clinic and familial protection can provide valuable support during the transitional period, with non-adherence reducing from 54% to 18% with attendance at an HIV support group, good parental monitoring/supervision and sufficient food [44].

Finally, while further implementation and operations research is urgently needed to better understand how to support young people and providers in achieving smooth transitions, in the interim, programme implementers may draw on the existing, albeit limited, guidance for transition in the context of other chronic conditions while working with partners to proactively build the evidence base needed to inform HIV-specific transition best practice.

## Conclusions

Only by moving from policy to practice through supportive policies and their implementation will we be closer to including adolescents in the 2030 goal of ending AIDS.

## List of abbreviations

AFHS  Adolescent-friendly health services

ALHIV Adolescents living with HIV

PMTCT Prevention of Mother-to-Child Transmission of HIV

SRH   Sexual and reproductive health
